# The PROBIT approach in estimating the prevalence of wasting: revisiting bias and precision

**DOI:** 10.1186/1742-7622-10-8

**Published:** 2013-08-27

**Authors:** Curtis J Blanton, Oleg O Bilukha

**Affiliations:** 1 International Emergency and Refugee Health Branch Centers for Disease Control and Prevention, 4770 Buford Hwy. MS-F-57, 30341 Atlanta, GA, USA

**Keywords:** Child Malnutrition, Wasting, Weight-for-Height, Z-score, Probit, Surveys

## Abstract

**Background:**

The PROBIT methodology was presented in the 1995 World Health Organization Technical Report on Anthropometry as an alternative to the standard prevalence based method of measuring malnutrition in children. Theoretically the PROBIT method will always give a smaller standard error than the standard prevalence method in measuring malnutrition. A recent article by Dale et al. assessed the PROBIT method for measuring global acute malnutrition measure and found that the method was biased and the precision was superior only for sample sizes less than 150 when compared to the standard method. In a manner similar to Dale, our study further investigated the bias and precision of the PROBIT method for different sample sizes using simulated populations.

**Results:**

The PROBIT method showed bias for each of the ten simulated populations, but the direction and magnitude of the average bias was changed depending on the simulated population. For a given simulated population, the average bias was relatively constant for all sample sizes drawn. The 95% half-width confidence interval was lower for the PROBIT method than the standard prevalence method regardless of the sample size or simulated population. The absolute difference in the confidence limits showed the most gains for the PROBIT method for the smaller samples sizes, but the ratio of confidence intervals was relatively constant across all sample sizes.

**Conclusions:**

The PROBIT method will provide gains in precision regardless of the sample size, but the method may be biased. The direction and magnitude of the bias depends on the population it is drawn from.

## Introduction

The measurement of population based estimates of malnutrition in children 6 to 59 months is a major component in assessing the nutrition levels in vulnerable populations. Typically, the prevalence of malnutrition is determined by taking a random survey sample of children from a population and indexing each selected child’s weight and height to the standard World Health Organization (WHO) Growth charts [1]. The resulting weight-for-height Z-scores (WHZ) are then compared to malnutrition thresholds; the number of children with WHZ less than the WHZ threshold is then counted and divided by the number of children in the sample. As a measure of precision, a 95% confidence limit around the estimate is calculated based on the sample size and sample design. This is the standard prevalence method of measuring malnutrition [1].

The 1995 WHO Technical Report 854 described the use of the PROBIT method to measure child anthropometry indicators in nutrition surveys as a more robust alternative to the standard prevalence-based approach [1]. The PROBIT method assumes the true distribution of WHZ comes from a normal distribution, so the distribution can be defined using the mean and the standard deviation (SD). Instead of counting the number of cases below a malnutrition WHZ threshold for a given sample, the PROBIT method estimates the prevalence of malnutrition indirectly by computing the area under the tail of the curve from -∞ to the threshold via the cumulative normal distribution function using the sample mean and standard deviation [2]. Alternatively, to reduce the influence of extreme values for a given survey, robust estimators of the sample mean, such as Winsorized or trimmed means, can be used instead of the standard sample mean [3]. The principal assumption around the PROBIT method when estimating the prevalence of wasting is that the WHZ follow a normal distribution.

The advantage of the PROBIT method is that it will, theoretically, always produce a smaller standard error around a prevalence estimate than the standard prevalence method because it assumes a normal distribution [2]. The other advantage is the method is less vulnerable to field measurement errors of a child’s height and weight versus the standard method which counts cases in the tail of the distribution. The disadvantage of the PROBIT method is that the bias of the PROBIT methodology depends upon the deviation of the true population of WHZ from the assumed normal distribution of WHZ defined by the mean and standard deviation. The prevalence based method on the other hand, assuming no measurement error, will give an unbiased estimate of the true prevalence.

Using a simulation based approach; a recent paper by Dale *et al*. examined the difference in bias and precision between the PROBIT method and the standard prevalence method for estimating the prevalence of global acute malnutrition (GAM) as well as severe acute (SAM) and moderate acute malnutrition (MAM) [4]. Ignoring clustering, they created simulated true populations by inflating field nutrition surveys and then drawing repeated random samples at different fixed sample sizes to calculate the precision and bias for each method. The results were aggregated across the simulated true populations at each of the fixed sample sizes. The Dale study found that the precision for the PROBIT methodology for GAM based on WHZ was superior to the standard method only for sample sizes <150. The paper also stated that PROBIT method overestimated the prevalence of GAM, MAM and SAM and one can correct for it with the simple subtraction of the bias.

After reviewing the Dale article, the authors had concerns about the generalizability of the reported aggregated results. One concern was that the results of the Dale study contradicted the theoretical model in which the precision from the PROBIT method should always be superior to the standard prevalence method irrespective of the sample size and not solely for sample sizes <150. The other problem was that the aggregation of the bias and precision for multiple simulated populations may have masked individual population differences. We suspected that bias would vary by population depending on WHZ fit to the normal distribution and the magnitude of the prevalence.

Our study investigated the discrepancy between theoretically expected results of precision for the PROBIT method vs. the standard method and the ones reported by Dale. In addition we examined the variation of the bias of the PROBIT method by simulated population. We first examined the difference in precision between the two methods assuming a population with a perfect fit to the normal distribution. Next, using simulated populations from inflated field surveys in a similar manner as Dale, we examined the bias and precision around prevalence of the WHO defined threshold of WHZ < −2 (wasting) instead of GAM which is defined as a WHZ < −2 or the presence of edema. We used wasting as opposed to GAM because we wanted to look at the behavior of the estimates without edema. Instead of aggregating across simulated populations, we looked at the precision and bias from individual simulated populations of different categories of quality and magnitudes of wasting.

## Methods

### Perfect fit to normal distribution

First, to illustrate a theoretical perfect fit of WHZ, the precision for each method was calculated assuming an imaginary distribution of WHZ that perfectly fit a normal population with a mean of 0.71 and SD = 1 - this results in a 10% prevalence estimate. For each method, the precision was calculated for 10 different sample sizes ranging from 50 to 900. The standard error (SE) for the prevalence method was calculated using the normal approximation to the binomial and the 95% Half-Width confidence interval (CI) was calculated by multiplying the SE*1.96. To calculate the 95% confidence intervals around the PROBIT estimate, the SE of the sample mean was calculated first along with 95% CI of the sample mean. Next, the cumulative normal distribution function was used to calculate the upper and lower bounds of the sample mean to obtain the confidence intervals around the PROBIT estimate.

### Dale et al. PROBIT estimation

To compare the standard method vs. the PROBIT method, Dale *et al*. created simulated population data sets using a database of 560 cluster survey datasets taking only records with WHZ between −5 and +5. Ignoring the cluster design, they inflated each dataset to a population of 17,000 using random sampling with replacement. A true prevalence was calculated for each of the 560 simulated population datasets by counting the cases of malnutrition and dividing by 17,000. From each population of 17,000, they drew 150 samples of 15 different sample sizes ranging from 50 to 500 stepped up by 25 each time. The prevalence was calculated for every sample using the standard prevalence method of counting cases and the PROBIT method with three different estimators of the mean and SD: 1) median and SD = 1; 2) mean and the observed SD; and 3) mean and observed SD using a Box-Cox transformed data to help normalize the data [5].

To measure the performance of the PROBIT methodology vs. the standard prevalence method, they examined both the bias and precision. The bias was measured by subtracting the estimate of GAM produced by each method from the true prevalence and then taking the mean (mean error). In the case of the standard method, the measure is an unbiased estimator of the percent GAM. The precision was measured by calculating the 95% limits of agreement (mean error ±1.96 * SD error) for each of the 15 sample sizes and populations separately. Summary measures of the bias and precision were calculated by taking the average across the 560 simulated populations at each of the 15 sample sizes.

### Simulations to assess the standard and PROBIT methods

To investigate the performance of PROBIT in the measure of wasting compared to the standard prevalence method, we used a total of 10 nutrition field surveys to create simulated populations of WHZ. We included four nutrition field surveys that were conducted by the US Centers for Disease Control and Prevention in Bhutanese refugee camps from 2007 through 2010 and used a simple random sampling design – these were considered good quality surveys with supervision at each stage of the survey process [6]. The other six nutrition surveys were randomly selected from a database of 390 surveys provided by Action Against Hunger International (ACF International) and the Food Security and Nutrition Analysis Unit for Somalia (FSNAU). The surveys were administered by non-governmental organizations, were conducted during 2001–2009, used cluster sampling design, had a sample size of at least 400, and were of variable data quality. To obtain a variety of simulation surveys, the surveys were separated into six categories by prevalence and quality prior to selection and one survey per cell was randomly selected (Table 1). The quality stratification was based on the SD of the WHZ as previously described [1].

**Table 1:** The number of surveys by prevalence and quality categories from the ACF international and FSNAU databases (N = 390)

	**Low Prevalence (Wasting < = 10%)**	**Medium prevalence (Wasting 10.1% to 20%)**	**High prevalence (Wasting >20%)**
High Quality SD* <1.1	3	48	28
Low Quality SD > =1.2	141	110	60

* SD, standard deviation.

For all surveys, the WHZ were based on the 2006 WHO Growth Standards [7] and were generated using the ENA software [8]. Extreme values were excluded from the analysis based on the WHO *flexible exclusion range* criteria, which defines an improbable value as a WHZ of < −4 and >4 from the observed mean [1].

In a manner similar to Dale, we created 10 simulated populations by inflating each of the surveys to a population of 20,000 using simple random sampling with replacement. From each simulated population, we drew 1,000 samples for each of 10 different sample sizes ranging from 50 to 500 incremented by 50. For each sample, we calculated the prevalence using the standard method of counting the number of children with a WHZ less than −2 and then the PROBIT method using the observed mean and SD. We also examined a robust estimator of the mean using Winsorization, but it was not superior to using the observed mean and SD; therefore, we have omitted those results from the article [3].

To compare the properties for the two different methods, we calculated the bias and precision for the 10 different sample size categories within each simulated population. The bias for each sample was calculated by subtracting the sample estimate of wasting prevalence from the true prevalence of the simulated population and then calculating average bias within each sample size category. The precision for each simulated population was estimated by calculating the half-width of the 95% CI (1.96 * SD) within each sample size category (n = 1000); the SD is the standard deviation of the sample prevalence estimates. Because the true population mean of each simulated population is a constant, the method we used to calculate the precision around the PROBIT estimate is mathematically equivalent to the 95% limits of agreement (mean error ±1.96 * SD error) ) used in the Dale article.

To examine the magnitude of the bias relative to the true value, the relative bias for the PROBIT was calculated for each simulation by dividing the average bias for a given sample size by the true prevalence value. To show the relative precision of the standard method to the PROBIT method, we calculated the ratio of the 95% half-width CI of the standard method to the PROBIT by dividing the average 95% half-width CI of the standard method by the average 95% half-width CI of the PROBIT.

To examine the deviations of the populations from the normal distribution we calculated the skewness, kurtosis, and the Shapiro-Wilk test for normality [9]. Significance for the Shapiro-Wilk test was set at p < 0.05.

The analysis for this paper was generated using Excel 2010 and SAS/STAT software, Version 9.3 of the SAS System for Windows [10].

## Results

### Perfect fit to normal distribution

The precision for PROBIT versus the standard method assuming a WHZ distribution that fits the normal distribution perfectly are presented in Table 2. The ratio of the half-width 95% CI of the standard method to the PROBIT was the same (1.7) regardless of the sample size. The half-width 95% CI for the PROBIT was smaller than standard prevalence for all the calculated sample sizes.

**Table 2:** The standard errors (SE) and confidence intervals (CI) for the PROBIT method versus the standard prevalence method assuming a WHZ perfect fit to the normal distribution (Mean WHZ = −0.72, SD = 1.0, prevalence = 10.0%)

**n**	**SE**	**SE**	**Half- Width 95% CI Standard prevalence method**	**Half Width of 95% CI PROBIT method**	**Ratio of Standard Prevalence CI to PROBIT CI**	**Absolute Difference of the 95% Half-Width CI PROBIT –Standard prevalence**
	**Standard prevalence method**	**PROBIT method**				
50	4.2%	2.5%	8.3%	4.9%	1.7	−3.4%
100	3.0%	1.8%	5.9%	3.5%	1.7	−2.4%
200	2.1%	1.2%	4.2%	2.4%	1.7	−1.7%
300	1.7%	1.0%	3.4%	2.0%	1.7	−1.4%
400	1.5%	0.9%	2.9%	1.7%	1.7	−1.2%
500	1.3%	0.8%	2.6%	1.5%	1.7	−1.1%
600	1.2%	0.7%	2.4%	1.4%	1.7	−1.0%
700	1.1%	0.7%	2.2%	1.3%	1.7	−0.9%
800	1.1%	0.6%	2.1%	1.2%	1.7	−0.9%
900	1.0%	0.6%	2.0%	1.1%	1.7	−0.8%

### Simulation results

The descriptive statistics for the original datasets (used as a basis for the simulations) are presented in Table 3. The WHZ SD’s were similar for the four Nepal surveys (range: 0.91-0.93). By definition, all of the low quality surveys had a SD > =1.2 and the high quality surveys <1.1. Based on the Shapiro-Wilk test for normality, all of the low quality surveys as well as three of the four Nepal surveys were significantly non-normal. All of the surveys had a small amount of skewness ranging from −0.17 and 0.31. The kurtosis was also relatively small ranging from 0.15 to 0.75. All of the surveys had very few or no subjects with edema.

**Table 3:** **Descriptive statistics of the nutrition surveys using weight-for-height Z (WHZ) scores for determining Wasting prevalence used for the simulation**^**a**^

**Data set**	**n**^**b**^	**Excluded**^**c**^	**GAM**^**d **^**Prevalence**	**Wasting Prevalence**	**Edemaprevalence**	**Mean WHZ**	**SD WHZ**	**Shapiro-Wilk**	**Kurtosis**	**Skewness**
								**p**		
Nepal 2007	497	0	4.2	4.2	0	−0.49	0.92	0.389	0.26	−0.16
Nepal 2008	502	0	9.2	9.2	0	−0.85	0.93	0.017	0.59	0.27
Nepal 2009	568	0	7.2	7.2	0	−0.76	0.91	0.034	0.75	−0.14
Nepal 2010	569	0	8.1	8.1	0	−0.65	0.91	0.006	0.51	0.05
Sudan North Darfur 2006–11 (high quality, high prevalence)	954	2	25.5	25.2	0.3	−1.37	1.02	0.131	0.26	−0.07
Sudan South Darfur 2007-9(high quality, medium prevalence)	802	3	19.1	19.0	0.1	−1.14	1.02	0.102	0.29	−0.17
Haiti (Artibonite) 2004–5 (high quality, low prevalence)	889	2	5.1	5.1	0	−0.25	1.04	0.817	0.27	−0.02
Somalia 2007(1)(low quality, high prevalence)	898	1	22.1	22.1	0	−1.03	1.29	<0.001	0.27	0.31
Somalia 2007(2) (low quality, medium prevalence)	905	0	10.3	10.2	0.1	−0.40	1.29	<0.001	0.15	0.13
Somalia 2006 (low quality, low prevalence)	905	14	9.3	9.1	0.2	−0.37	1.23	0.005	0.30	0.19

^a^Excludes observations < −4 WHZ or >4 WHZ from observed mean, ^b^Observations used in the analysis, ^c^Number of observations that were excluded (<−4 WHZ or >4 WHZ from observed mean), ^d^GAM is defined as WHZ < −2 or the presence of edema.

The bias for the PROBIT methods changed in magnitude and direction depending on the simulation survey used, but the magnitude of the bias for a given simulation dataset was relatively constant regardless of the sample size (Table 4). For 8 of the 10 surveys the bias was positive. The bias was the highest relative to the true prevalence of wasting for the 2007 through 2010 Nepal surveys (Table 5).

**Table 4:** Average bias for the PROBIT method by simulation survey

**n (Sample Size)**	**Nepal 2007**	**Nepal 2008**	**Nepal 2009**	**Nepal 2010**	**Sudan 2006**	**Sudan 2007**	**Haiti 2004**	**Somalia 2007(1)**	**Somalia 2007(2)**	**Somalia 2006**
50	1.09	1.57	1.37	−1.16	1.32	0.87	−0.15	0.84	0.45	0.35
100	0.88	1.40	1.25	−1.21	1.41	0.91	−0.16	0.70	0.34	0.38
150	0.87	1.29	1.37	−1.24	1.52	1.04	−0.25	0.73	0.57	0.33
200	0.90	1.32	1.37	−1.23	1.48	0.99	−0.18	0.76	0.45	0.42
250	0.84	1.29	1.27	−1.23	1.46	1.04	−0.22	0.70	0.46	0.31
300	0.90	1.36	1.34	−1.30	1.44	1.13	−0.26	0.79	0.52	0.39
350	0.87	1.40	1.29	−1.25	1.43	1.10	−0.26	0.86	0.53	0.39
400	0.88	1.44	1.32	−1.29	1.36	0.98	−0.25	0.76	0.50	0.39
450	0.92	1.39	1.29	−1.29	1.42	1.07	−0.26	0.71	0.54	0.36
500	0.86	1.39	1.28	−1.26	1.45	1.03	−0.29	0.79	0.52	0.34

**Table 5:** Relative Bias (bias/true estimate) of PROBIT methodology by simulation survey

**n (Sample Size)**	**Nepal 2007**	**Nepal 2008**	**Nepal 2009**	**Nepal 2010**	**Sudan 2006**	**Sudan 2007**	**Haiti 2004**	**Somalia 2007(1)**	**Somalia 2007(2)**	**Somalia 2006**
50	0.26	0.17	0.19	−0.14	0.05	0.05	−0.03	0.04	0.04	0.04
100	0.21	0.15	0.18	−0.15	0.06	0.05	−0.03	0.03	0.03	0.04
150	0.21	0.14	0.19	−0.15	0.06	0.06	−0.05	0.03	0.06	0.04
200	0.21	0.14	0.19	−0.15	0.06	0.05	−0.04	0.03	0.04	0.05
250	0.20	0.14	0.18	−0.15	0.06	0.06	−0.04	0.03	0.04	0.04
300	0.22	0.15	0.19	−0.16	0.06	0.06	−0.05	0.04	0.05	0.04
350	0.21	0.15	0.18	−0.16	0.06	0.06	−0.05	0.04	0.05	0.05
400	0.21	0.16	0.18	−0.16	0.05	0.05	−0.05	0.03	0.05	0.05
450	0.22	0.15	0.18	−0.16	0.06	0.06	−0.05	0.03	0.05	0.04
500	0.21	0.15	0.18	−0.16	0.06	0.05	−0.06	0.04	0.05	0.04

**Figure 1:**
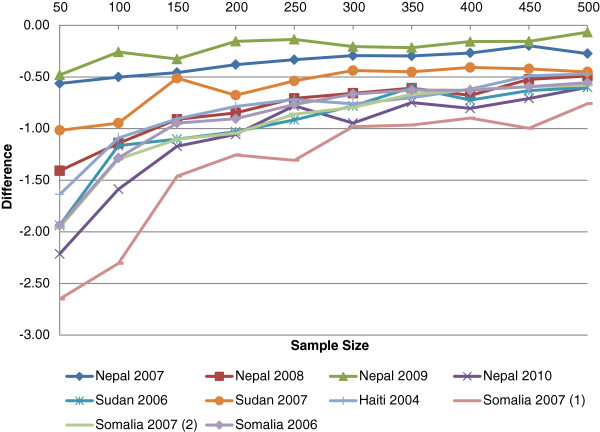
The absolute difference in the 95% half-width confidence limit between the PROBIT method - standard prevalence method.

**Figure 2:**
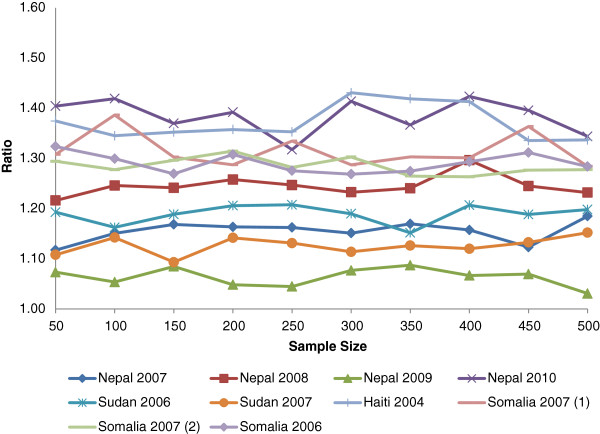
The ratio of standard prevalence method 95% half-width CI to the PROBIT method 95% half-width CI.

The precision, represented by the half-width 95% CIs, was superior for the PROBIT method for all the simulation datasets and sample sizes (Table 6). The absolute difference in the CI shows the largest absolute gains in precision for the smaller sample sizes (Figure 1). Within each simulation survey, the ratio of the half-width CI of the standard method to the PROBIT method was relatively constant across all sample sizes for each simulation database (Figure 2). These results are consistent with the theoretical model for precision mentioned above.

**Table 6:** 95% Half-Width confidence intervals for the standard and PROBIT method

**n**	**Method**	**Nepal 2007**	**Nepal 2008**	**Nepal 2009**	**Nepal 2010**	**Sudan 2006**	**Sudan 2007**	**Haiti 2004**	**Somalia 2007(1)**	**Somalia 2007(2)**	**Somalia 2006**
50	Standard	5.4	7.9	7.0	7.7	12.1	10.9	5.9	11.5	8.6	7.9
	PROBIT	4.8	6.5	6.6	5.5	9.7	9.0	4.0	8.4	6.7	5.9
100	Standard	3.8	5.8	5.1	5.4	8.4	7.8	4.1	8.2	6.0	5.6
	PROBIT	3.3	4.6	4.8	3.8	6.8	6.3	3.0	6.0	4.7	4.1
150	Standard	3.2	4.7	4.2	4.3	6.8	6.2	3.5	6.3	4.8	4.3
	PROBIT	2.7	3.8	3.9	3.2	5.4	5.2	2.5	4.7	3.7	3.2
200	Standard	2.7	4.1	3.4	3.8	5.9	5.4	3.0	5.6	4.4	3.9
	PROBIT	2.3	3.3	3.2	2.7	4.7	4.5	2.1	4.1	3.3	2.8
250	Standard	2.4	3.6	3.2	3.2	5.5	4.6	2.8	5.2	3.9	3.4
	PROBIT	2.1	2.9	3.0	2.5	4.4	3.9	1.9	3.9	3.0	2.5
300	Standard	2.2	3.5	2.9	3.2	4.8	4.3	2.5	4.8	3.4	3.1
	PROBIT	1.9	2.8	2.7	2.3	3.8	3.5	1.7	3.5	2.6	2.3
350	Standard	2.0	3.1	2.7	2.8	4.5	3.9	2.3	4.2	3.2	2.9
	PROBIT	1.7	2.5	2.5	2.0	3.6	3.3	1.6	3.1	2.5	2.1
400	Standard	2.0	3.0	2.5	2.7	4.3	3.8	2.1	3.9	3.0	2.7
	PROBIT	1.7	2.3	2.4	1.9	3.3	3.2	1.5	3.0	2.4	2.0
450	Standard	1.8	2.7	2.4	2.5	4.0	3.6	2.0	3.8	2.7	2.5
	PROBIT	1.6	2.1	2.2	1.8	3.2	3.0	1.4	2.9	2.1	1.9
500	Standard	1.8	2.6	2.2	2.3	3.8	3.5	1.9	3.5	2.7	2.5
	PROBIT	1.5	2.1	2.2	1.7	3.0	2.8	1.3	2.7	2.1	1.9

## Discussion

We have shown that the magnitude and direction of the bias for the PROBIT methodology depended on the simulation survey dataset in contrast to the Dale article which stated that the PROBIT method overestimates GAM and can be corrected with a uniform value. Our bias was also consistently higher than those reported in the Dale article, but this may be due to the fact that they averaged the bias over 560 surveys and the positive and negative values then cancelled each other out. The relative bias was higher for the Nepal simulation datasets, which all had lower SD (<1) and a higher kurtosis than the other six datasets. On the other hand, all three Somalia datasets that were significantly non-normal by Shapiro-Wilk test showed much lower relative bias. Further analysis is needed to determine if the variation in the magnitude and direction of the bias can be explained by the shape of the WHZ distribution and the degree of non-normality. As suggested in the Dale article, a transformation to make the data more normal may reduce the bias.

Our results show the PROBIT method consistently had higher precision around the prevalence of wasting than the standard method. This was true for the perfect fit to the normal distributions and our simulation results. Our PROBIT method simulations yielded lower half-width CI than the standard method for estimating wasting regardless of the sample size. This is consistent with the theoretical model but is a different result than presented in the Dale article, which concluded that the width of the 95% limits of agreement for the PROBIT methods were superior only for sample sizes <150. Certainly though, the largest absolute gains in precision were for the lower sample sizes. We do not think the difference in precision between our results and Dale’s is due to due to the different simulation data sets used for each study. Our precision results were consistently lower for the PROBIT method despite having intentionally selected surveys of varying quality and prevalence sizes. A possible reason for the discrepancy in the results could be the manner in which Dale et al. aggregated their results when calculating the precision around their estimates. It appears they may have calculated the standard deviation of the mean error for all samples from all the simulated populations. Calculating the precision in this manner would artificially decrease the precision of the PROBIT method because the magnitude and directionality of the bias depends on the simulated population. The standard method would not be affected theoretically because it is an unbiased estimate.

The results of our study are limited by the fact that our analyses used simulated WHZ population data created from sample survey datasets instead of the actual exhaustive population data. We do not know if the true populations would have had distributions closer or farther away from the normal distribution. Also, the influence of measurement error in the field was ignored; this may have caused more bias for the standard prevalence method than the PROBIT method. Another limitation is that we ignored the cluster survey design of the non-Nepal surveys when we created a simulated population of WHZ using simple random sampling. Further investigation is needed to determine the behavior of the PROBIT method when applied to cluster surveys. Also, we looked only at wasting, which does not include edema. Looking at GAM in the populations where edema is high and many of the edema cases have WHZ greater than −2, would add an additional positive bias, since edema cases are not accounted for directly by WHZ distribution.

## Conclusions

The results of our study clearly show that the bias from PROBIT method is population dependent and should not be generalized for all populations. In our examples, the PROBIT method consistently outperformed the standard method in terms of precision for all sample sizes as opposed to the Dale article which stated the gain in precision is only for small sample sizes. We agree with the Dale article that the PROBIT method is most advantageous for the smaller sample sizes, where the gains in absolute precision are the greatest. However, the ratio of the precision of the standard method to the PROBIT method was shown to be close to constant across sample sizes, consistent with the theoretical model. Further work is needed to explore whether some of the survey data characteristics (e.g., SD, skewness, kurtosis, prevalence, etc.) can consistently explain the degree and directionality of bias, so caution should be used when applying this method.

## Competing interests

Both authors declare that they have no competing interests.

## Authors’ contributions

CJB and OOB designed the study, CJB conducted the analysis, and CJB and OOB drafted the manuscript. Both authors read and approved the final manuscript.
